# Seasonality of birth outcomes in rural Sarlahi District, Nepal: a population-based prospective cohort

**DOI:** 10.1186/1471-2393-14-310

**Published:** 2014-09-06

**Authors:** Michelle M Hughes, Joanne Katz, Luke C Mullany, Subarna K Khatry, Steven C LeClerq, Gary L Darmstadt, James M Tielsch

**Affiliations:** Department of International Health, Bloomberg School of Public Health, Johns Hopkins University, 615 North Wolfe Street, Baltimore, Maryland 21205 USA; Nepal Nutrition Intervention Project–Sarlahi, Kathmandu, Nepal; Global Development Division, Bill and Melinda Gates Foundation, Seattle, WA USA; Department of Global Health, Milken Institute School of Public Health, George Washington University, Washington, DC USA

**Keywords:** Gestational age, Low birth weight, Intrauterine growth restriction, Neonatal mortality, Season, Preterm birth

## Abstract

**Background:**

While seasonality of birth outcomes has been documented in a variety of settings, data from rural South Asia are lacking. We report a descriptive study of the seasonality of prematurity, low birth weight, small for gestational age, neonatal deaths, and stillbirths in the plains of Nepal.

**Methods:**

Using data collected prospectively during a randomized controlled trial of neonatal skin and umbilical cord cleansing with chlorhexidine, we analyzed a cohort of 23,662 babies born between September 2002 and January 2006. Project workers collected data on birth outcomes at the infant’s household. Supplemental data from other studies conducted at the same field site are presented to provide context. 95% confidence intervals were constructed around monthly estimates to examine statistical significance of findings.

**Results:**

Month of birth was associated with higher risk for adverse outcomes (neonatal mortality, low birthweight, preterm, and small for gestational age), even when controlling for maternal characteristics. Infants had 87% (95% CI: 27 – 176%) increased risk of neonatal mortality when born in August, the high point, versus March, the low point.

**Conclusion:**

Seasonality of neonatal deaths, stillbirths, birth weight, gestational age, and small for gestational age were found in Nepal. Maternal factors, meteorological conditions, infectious diseases, and nutritional status may be associated with these adverse birth outcomes. Further research is needed to understand the causal mechanisms that explain the seasonality of adverse birth outcomes.

**Electronic supplementary material:**

The online version of this article (doi:10.1186/1471-2393-14-310) contains supplementary material, which is available to authorized users.

## Background

There is a well-documented relationship between seasonality and poor birth outcomes, which varies across populations and geographic regions [1–3]. Various hypotheses have been examined to understand the causal mechanisms for seasonality of birth outcomes including maternal factors [4], meteorological conditions [5], infection patterns [6], and access to appropriate nutrition [7].

The majority of these studies have been conducted in high-income countries with relatively few from low- and middle-income countries where the effects of these factors may be greater [4, 7–10]. In Bangladesh [8], September through November carried the highest risk of being born low birth weight. Despite the plethora of published data on seasonality, little is known about the seasonal variations of neonatal mortality, stillbirth, low birth weight, prematurity, and small for gestational age (SGA) in rural South Asia.

This study aims to provide a descriptive analysis of seasonality of birth outcomes over an approximately three-year period in the rural plains of Nepal. In spite of progress made by Nepal in meeting its Millennium Development Goal 4 for child mortality, adverse pregnancy outcomes and early child mortality are still high [11]. Examination of the seasonality of these adverse events may improve our understanding of the risk factors associated with these events, and which are in the causal pathway. Seasonal patterns may also help inform interventions or the optimal timing of these interventions in community settings.

## Methods

### Population

The Nepal Nutrition Intervention Project study area is based in Sarlahi district, a rural community in the low-lying plains of Nepal. The data were collected as part of a previously published community-based, cluster-randomized, placebo-controlled trial of chlorhexidine skin and umbilical cord cleaning on neonatal morbidity and mortality conducted from September 2002 to January 2006 [12, 13]. Briefly, incident pregnancies were identified through monthly home visits to all households in the Sarlahi study area. Pregnant women were enrolled and their infants were randomized, by geographic sector, to receive either placebo skin cleansing or chlorhexidine 0.25% skin cleansing as soon as possible after birth [12]. Nested within this main trial was a second study of umbilical cord care where within each skin care group (0.25% chlorhexidine or placebo) infants were randomized to receive either 4% chlorhexidine, soap/water, or dry cord care only [13]. This study was approved by the Nepal Health Research Council and the Committee on Human Research of the Johns Hopkins Bloomberg School of Public Health.

### Data collection

Data were prospectively collected on neonatal mortality, stillbirths, birth weight, and gestational age. Local female health care workers visited all households in the study area monthly in order to identify, consent and enroll incident pregnancies (at approximately ~6 months gestation). All enrolled women received 90 days of iron-folic acid supplements, weekly vitamin A supplementation, deworming, and a clean delivery kit, and women for whom tetanus vaccination was indicated were offered the vaccine. Project workers visited the households (~92% were home births) as soon as possible after delivery for a maternal and newborn health assessment. Project workers visited households a total of ≤11 times (days 1–4, 6, 8, 10, 12, 14, 21, and 28) to assess infant morbidity and mortality. Infants were weighed as soon as possible after birth using a digital scale (Seca DigitalBaby Scale Model 727) that measured weight to within 2 grams. Women were asked to report the month of pregnancy at the time of enrollment; soon after delivery they were asked again to estimate the length of the pregnancy in months. These two separate reports both yielded estimates of gestational age at delivery, which were then averaged to estimate the final gestational age for analysis.

We obtained daily maximum and minimum temperature recordings (in Celsius) from Simra (27°09’34"N, 84°58’47" E) and Janakpur (26°42’39" N, 85°55’27" E) airports, which are approximately 52 km east and 60 km west, respectively, of the geographical center of the study area (data courtesy of the Government of Nepal, Department of Hydrology and Meteorology, Ministry of Environment, Science, and Technology) [14]. Data on food security were obtained from a follow-up study conducted in the same population from September 2006 to March 2008. Food insecurity was defined as the percent of households who reported no rice stored for consumption by household members.

### Data analysis

We present descriptive analyses of birth outcomes by month. Neonatal and perinatal mortality was anchored on month of birth (i.e. a birth in January but death in February was classified as a January death with live births in January as the denominator for the mortality rate). Births were considered stillbirths if the mother reported that her infant neither cried, moved, nor breathed following delivery, and gestational age was 28 weeks or greater. The stillbirth rate was calculated as the number of stillbirths over the total number of live births plus stillbirths (multiplied by 1000). Infants were categorized into term (≥37 weeks), preterm (<37 weeks), and very preterm (<34 weeks) gestational age categories based on completed weeks of gestation. Birth weights were included only if collected within 72 hours of birth. Infants were categorized into three birth weight categories: (1) normal weight ≥2500 grams (g), (2) low birth weight <2500 g, and (3) very low birth weight <2000 g. Two sex-specific analytic methods were used to identify infants who were SGA: the 10th percentile cut-off described by Alexander [15] and the 3rd percentile cut-off describe by Oken [16]. Poisson exact 95% confidence intervals were constructed around the monthly birth estimates. Binomial exact 95% confidence intervals were constructed around birth outcome estimates by month. Logistic regression models were used for our main outcomes (neonatal mortality, preterm, low birthweight, and SGA) with month, modeled as a dummy variable, as the main exposure of interest. The models were adjusted for infant sex, maternal literacy, parity, ethnicity, and socio-economic status to control for their association with birth outcomes. This study was prepared and presented according to the STROBE guidelines for observational studies (Additional file 1). Analyses were conducted with Stata version 12.1.

## Results

**Figure 1:**
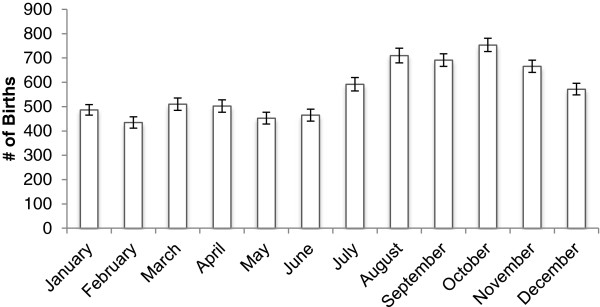
**Average number of births by month, September 2002 to January 2006, Sarlahi District, Nepal.**

23,662 infants were enrolled from September 2002-January 2006 (Figure 1, Additional file 2: Table S1). The refusal rate for infants’ participation was 0.02% [12]. The number of births by year during the study period was 2539 in 2002 (September-December only), 6998 in 2003, 6939 in 2004, 6834 in 2005, and 352 in 2006 (January only). Observing no strong differential patterns for months across years, we combined the monthly estimates for all available years with data. The sex distribution at birth favored boys (51.5%). A higher number of births took place from July through December with October being the peak birth month (753 births, 95% CI: 727 – 781) compared to February with the lowest average births (434, 95% CI: 411 – 459).

**Figure 2:**
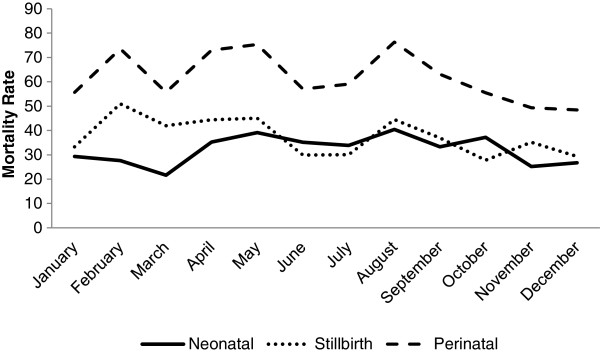
**Average stillbirth, perinatal, and neonatal mortality rates by month, September 2002 to January 2006, Sarlahi District, Nepal.**

The overall neonatal mortality rate was 32.1 deaths per 1000 live births (95% CI: 29.9 – 34.4) with a substantial monthly variation [range: 21.6 – 40.4] (Figure 2, Additional file 2: Table S1). Neonatal mortality was highest between April and October. In the peak month of August, neonatal mortality was almost double that in the lowest mortality month of March (Risk Ratio 1.87, 95% CI: 1.27 – 2.76). Stillbirths had a different trend than the other adverse birth outcomes with the highest and lowest stillbirth rates observed in February (51.0 per 1000, 95% CI: 40.0 – 64.0) and October (27.8 per 1000, 95% CI: 22.2 – 34.2), respectively. Three peaks were observed for perinatal mortality in February, April-May, and the highest in August (76.3 deaths per 1000 births, 95% CI: 65.6 – 88.1); the lowest perinatal mortality occurred in November and December (48.4 deaths per 1000 births, 95% CI: 22.9 – 36.9).

**Figure 3:**
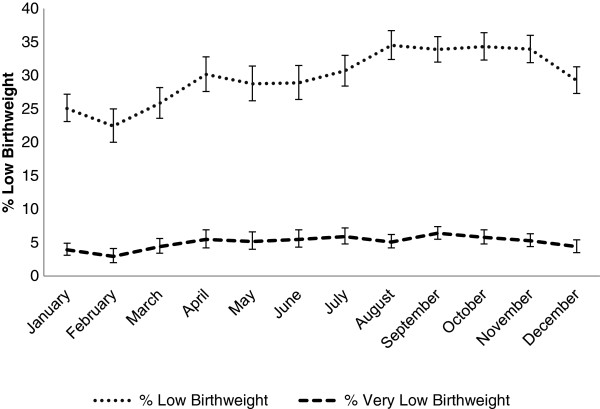
**Percent low birthweight categories by month, September 2002 to January 2006, Sarlahi District, Nepal.**

Birthweights were missing for 14.5% of all births (11% collected >72 hours, 2% never collected, 1.5% deaths, and <1% refusal). The prevalence of low birth weight (<2500 g) and very low birth weight (<2000 g) was 30.5% (95% CI: 29.9 – 31.1) and 5.1% (95% CI: 4.8 – 5.4), respectively (Figure 3, Additional file 3: Table S2). Infants had higher prevalence of low birth weight from August through November with a peak prevalence in August of 34.5% (95% CI: 32.4 – 36.7). Infants born between January and March were least likely to be low birth weight, with the lowest prevalence in February (22.4%, 95% CI: 20.0 – 25.0). There was a similar pattern for very low birth weight infants with the prevalence in February (2.9%, 95% CI: 2.0-4.1) statistically significantly lower than the prevalence between June and November. The peak very low birth weight prevalence was in September (6.4%, 95% CI: 5.5-7.4). The mean birth weight for this population was 2687 g (95% CI: 2681 – 2694), and was highest in February (2773 g, 95% CI: 2749 – 2798) and lowest in October (2644 g, 95% CI: 2626 – 2662). Males had higher average weight than females (2735 vs. 2637 g, difference = 98.0 g [95% CI: 86.3 – 109.8]) (Additional file 4: Figure S1).

**Figure 4:**
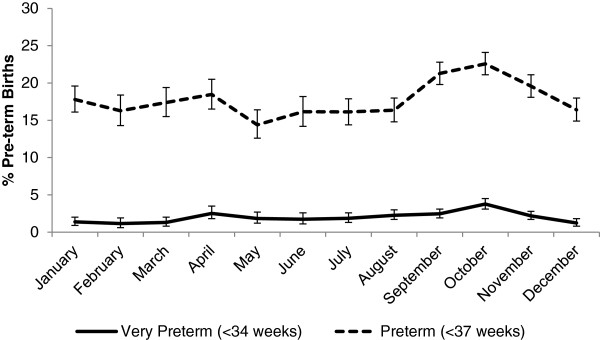
**Percent preterm by month, September 2002 to January 2006, Sarlahi District, Nepal.**

Overall 18.3% (95% CI: 17.8 – 18.8) of births were preterm (<37 weeks) (Figure 4, Additional file 5: Table S3). The prevalence of preterm ranged from 14.4% (95% CI: 12.6 – 16.4) in May to 22.6% (95% CI: 21.1 – 24.1) in October. The period of highest prevalence of preterm birth was between September and November and the lowest risk was between May and July. The average gestational age was lowest in October (38.8, 95% CI: 38.7 – 38.9) and highest in July (39.6, 95% CI: 39.5 – 39.7) (Additional file 6: Figure S2).

**Figure 5:**
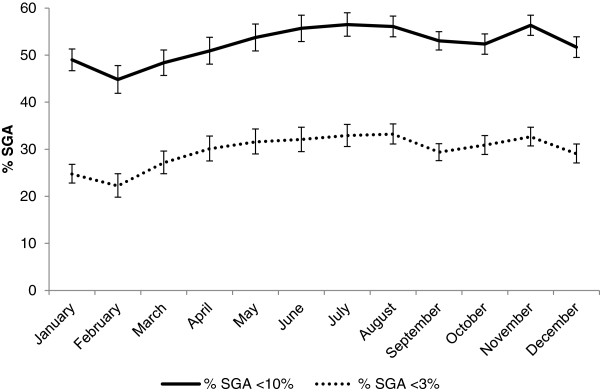
**Percent small for gestational age by month, September 2002 to January 2006, Sarlahi District, Nepal.**

The month-specific percent SGA averaged 52.7% (95% CI: 52.0 – 53.4) and 29.9% (95% CI: 29.2 – 30.5) using the <10% and <3% criteria, respectively (Figure 5, Additional file 7: Table S4). The two definitions we used for SGA yielded similar seasonal patterns. Infants were least likely to be SGA <10% between January and March, similar to the pattern for low birth weight. Infants were most likely to be SGA between June and August and in November. SGA <10% ranged from 44.8% (95% CI: 41.9 – 47.8) in February to 56.4% (95% CI: 54.2 – 58.5) in November.

The percent SGA and preterm averaged 4.5% (95% CI: 4.2 – 4.8) and 1.4% (95% CI: 1.3 – 1.6) using the <10% and <3% criteria, respectively. The percent SGA (<10%) and preterm ranged from a low of 3.2% (95% CI: 2.3- 4.3) in May to 6.0% (95% CI: 5.0 – 7.1) in October. Infants had the highest risk of being preterm and SGA (<10%) in September and October (Additional file 8: Table S5 and Additional file 9: Figure S3). The months of lowest risk of being both preterm and SGA (<10%) were January to March and May.

While we do not have data from this Nepal cohort on household food security by month we do have food security data from another cohort in the same study area from September 2006 to March 2008. Food insecurity was highest in May to June and lowest from October to April (Additional file 10: Figure S4). We also show the monthly average maximum and minimum temperatures as a reference to temperature exposures during different months of pregnancy (Additional file 11: Figure S5). Women and infants experienced the highest temperatures in April through September and the lowest temperatures in December to February. Recent (2012) data (unpublished) from the Nepal study area shows a seasonality of RSV infection with peaks in September to December. The peak of the influenza season runs from August through October [17]. A summary of the seasonality of birth outcomes and potential risk factors is provided in Additional file 12: Table S6.

We used a logistic model for neonatal mortality, preterm, low birthweight, and SGA to determine if the monthly differences remain upon adjustment for infant sex and maternal factors of literacy, parity, ethnicity, and socioeconomic status (Additional file 13: Table S7). Adjusting for infant sex and maternal factors had no statistical impact on the difference in birth outcomes by month.

## Discussion

These data demonstrate statistically significant seasonal variations in births and adverse birth outcomes in Nepal. The observed monthly pattern is likely due to month of year serving as a proxy for other factors causally associated with adverse birth outcomes. The seasonal pattern is not uniform across birth outcomes, likely reflecting different etiologies and time frames of risk for each of the examined birth outcomes. Stillbirths were in general highest in the hot and dry season (February – May) while neonatal mortality was increased from the hot and dry season through to post-monsoon season (April – October). Low birthweight and SGA had a similar pattern with increased prevalence from monsoon to post-monsoon (August – November), while high preterm prevalence was primarily during post-monsoon (September to November).

### Maternal factors

One theory postulated for seasonality of birth outcomes is that mothers with different socioeconomic and other characteristics preferentially marry and give birth at different times of the year [9, 18–21]. However, recent data from Currie finds a persistent seasonality even when controlling for maternal factors using a within-mother analysis [6], which is a similar finding from Torche and our study [21]. Two ethnic groups predominate in the Nepal study region, the *Pahadi* (hills) and the *Madeshi* (plains) [22]. The *Pahadi* are in general better off economically and have better health and nutritional status than the *Madeshi.* The *Madeshi* are more likely to give birth in April to May and August to November (data not shown) than the *Pahadi*. Literacy has a similar pattern with illiterate women more likely to give birth in March to May and September-December. Women from the lowest socioeconomic quartile are also most likely to give birth in April and September to December. These categories overlap with each other and also correspond with the pattern of higher prevalence of preterm birth and low birth weights in April and August to November. Despite these differences in birth timing by maternal characteristics, our model shows that controlling for these factors does not statistically significantly change the monthly patterns observed in birth outcomes.

### Food insecurity

Food insecurity has also been shown to be associated with poor birth outcomes [1]. A study in Bangladesh found that low birth weights were correlated with seasonal food availability and poor maternal nutrition [8]. In the Gambia it was hypothesized that SGA was associated with the hungry season and prematurity was associated with increased agricultural labor [9]. In a randomized control trial from the Gambia, villages that received maternal nutrition supplementation (20 weeks before delivery) showed a reduced seasonality of low birth weight indicating the importance of seasonality of food availability in impacting birth weights [7]. We found that the highest prevalence of low birth weight occurs during August to November, associated with the highest food insecurity (May to June) for this cohort during the 2nd and 3rd trimesters when fetal growth is accelerating. Data from a previous study in the area show maternal anemia to be highest (June to November) in the seasons corresponding to lowest birth weights [23]. Several mechanisms may underlie this relationship including iron deficiency’s stimulation of corticotropin releasing hormone, which increases fetal cortisol, which in turn inhibits the growth of the fetus [24]. The period of highest neonatal mortality is from April to October, encompassing the highest period of food insecurity.

### Environmental conditions

Factors related to the environment including temperature, humidity, and sunlight have been associated with birth outcomes [1, 21, 25–35]. Mannan found that severe disease in infants, likely associated with neonatal mortality, was associated with higher temperatures and the heat humidity index [36]. Our findings are consistent with this as the highest neonatal mortality period is within the hot season (April to September). Neonatal hypothermia occurs during all seasons in Nepal, but it does vary seasonally with higher prevalence in the colder winter months; however, this likely is a small contribution to mortality as the coldest months have the lowest mortality [14]. Parental attention to warming measures is increased in these colder months, mitigating the adverse effects of decreased temperature [14].

Other investigators have found a risk for preterm births when mothers were exposed to high ambient temperature or high heat-humidity index near the time of delivery [25, 28, 31, 32]. However, Porter found no increased risk of premature birth when mothers were exposed to short term heat stress [27]. Our data show the highest preterm births correspond with high temperature exposure in the 2nd or 3rd trimester. One biological explanation, based on animal models, for increased preterm risk is that high temperatures elevate the chance of dehydration [37]. This leads to greater risk of labor induction due to decreased uterine blood flow and increased prostaglandin and oxytocin production.

The literature regarding temperature and low birth weight is less consistent. Etler and Murray found low temperatures in the 2nd trimester were associated with low birth weight [26, 29]. A study from Scotland found that low temperatures in the 1st trimester were associated with decreased birth weight while high temperatures in the 3rd trimester were associated with increased birth weight [33]. Flouris and Torche reported lower birth weight was correlated with lower temperatures at the time of birth [21, 32], while Tustin found no such association [30]. The Nepal data show that the prevalence of low birth weight is highest for fetuses that experienced cooler temperatures in the 1st trimester with increasing temperatures during the 2nd trimester but this may be confounded with maternal characteristics, nutritional status and/or infection.

Tustin found that exposure to high levels of sunshine during the 1st trimester was associated with increased birth weight, postulating that light exposure increases levels of insulin-like growth factor, important for fetal growth [30]. Torche also found that increased exposure to sunlight in the 1st and 3rd trimesters was associated with increased birth weight [21]. Weber found that height at age 18 is dependent on month of birth using an Austrian male cohort and postulated that melatonin may play a role in growth [35]. Another hypothesis is that the effect of sunshine on vitamin D levels may affect fetal growth [34] and risk of preterm delivery [38]. A previous study in Sarlahi, Nepal, found the lowest levels of Vitamin D deficiency in June through August (4.3% prevalence) [23], supporting a hypothesis that 1st trimester exposure to sunlight (low Vitamin D deficiency) is correlated with high weights at birth (March through May). However, birth weights in our study peak in January and February, months that do not correspond with low levels of Vitamin D deficiency (24.4% prevalence during December through February [23]).

### Infectious diseases

One recent US study used a within-mother analysis to control for maternal factors and found a persistent seasonal pattern of birth outcomes with the highest risk for preterm birth in January and February [6]. The authors hypothesize that this may be related to adverse effects from influenza given that the peak of the influenza season corresponds to when fetuses are nearing full-term. Our data support this hypothesis, showing a similar peak of preterm and low birth weight births (September to November) overlapping the peak of the influenza season (August to October) in Nepal [17].

One study from the Gambia suggests that a peak in preterm births in October is associated with peak time for malaria infections although the evidence is not conclusive [9]. A previous study showed 20% prevalence of parasitemia (*Plasmodium vivax* only) with no clinical malaria so this is unlikely to play an important role in adverse birth outcomes as it might in Sub-Saharan Africa [39]. Another earlier study in the same area found that early summer (May and June) has the highest maternal diarrheal prevalence [23], which may lead to poorer maternal nutrient absorption, impacting SGA and low birth weight prevalence. Hygiene practices, including menstrual and post-defecation, may also vary by month increasing the risk of intrauterine infection, which is associated with preterm births [40]. Maternal sexually transmitted infections have also shown seasonal patterns and been associated with preterm delivery and mortality [41]. Data from our area collected prior to the current study suggested low prevalence of two sexually transmitted infections, *Neisseria gonorrhoeae (1.0%)* and *Chlamydia trachomatis (2.3%)* [42]. The impact of these infections on adverse outcomes would be expected to be minimal given their low prevalence in the study population, although these data come from a period 2 years before our study period.

### Strengths and limitations

As with all ecological associations, this study cannot identify a causal link between season of birth and poor birth outcomes. While we used regression to model seasonality of outcomes, controlling for maternal characteristics, this was not an exhaustive model and was missing potential factors, which were unmeasured in our study such as maternal infections and nutritional deficiencies. A second limitation is that we cannot isolate the true period or periods of exposure that are causally linked to various adverse birth outcomes. For example, the relative importance of exposure(s) immediately preceding the birth or accumulated exposure over different gestational periods is unknown [43]. Another limitation is that we only have data from similar, more recent studies, regarding respiratory virus transmission, food security, and vitamin D deficiency.

Our ascertainment of gestational age is based on women’s recall of last menstrual period, which may lead to misclassification of preterm. Our population estimates of preterm and SGA, however, are similar to those found in a previous study at our field site that used more sensitive gestational age measures [44]. We expect the measurement error to lead to non-differential misclassification and likely reduce the seasonal effects seen, not bias the estimates in a specific direction. There may have been misclassification of stillbirths and neonatal mortality but we have included perinatal mortality to help assess the difference in trends.

As our study was nested within a randomized controlled trial, there was some impact on neonatal outcomes from iron supplementation [45] (distributed to all) and the interventions delivered to randomization groups (skin and umbilical cord cleaning) [12, 13]. Any reduction in mortality would likely reduce the seasonality differences observed. Further, the provision of clean delivery kits and iron supplementation are a part of Nepal public health policy even if carried out unevenly; chlorhexidine for cord care is part of this current package as a result of the trial. The results seen are therefore indicative of seasonal differences in the presence of effective policy implementation.

One source of missing birthweight data was from infants who died too soon after birth and did not have an opportunity for weighing (1.5% of all births). Given the reverse J-shape of the mortality curve, these infants who died soon after birth would likely be lower birthweight (if preterm) or higher birthweight (thus at increased risk for birth asphyxia due to obstructed labor) compared to infants who did not die soon after birth [46]. In our study, infant deaths contributed to only 10% of missing birthweight data so this bias likely has a small effect. The major source of missing birthweight data was lack of weighing before 72 hours (74% of missing birthweights). There were some differences in prevalence of missing weights due to collection >72 hours by month (range 5-18% missing of all births). This variation could bias our birthweight estimates by month if the differences in percentages collected >72 hours are also related to factors associated with low birthweight. We saw higher percentage of missing data in October (18%) and November (15%), which are months where the prolonged holidays of *Daishan* and *Tihar* are observed. Since the primary reason for missing weights in this time period was fewer workers available to collect birthweight, there is no reason to believe this biased the seasonal variations. In a previous paper using data from our site we looked at the effect of missing birthweight data and imputation to understand how this may affect results [46]. The previous study compared effect estimates using crude and imputed birthweight data and did not find a significant difference between results, indicating that the bias from excluding these birthweights is not significant. Examining differences in infant and maternal characteristics by whether birthweight was missing showed some statistically significant differences but the absolute differences were small (Additional file 14: Table S8).

Strengths of this work are that it is a large, prospective, population-based study that is representative of South Asian populations for which we have birth weight, gestational age and survival outcomes. With a refusal rate of less than 1% and the sampling of all pregnant women in the study area the data are highly representative of Sarlahi District. Sarlahi District, is located in the *terai* region, where the majority of the Nepalese population lives and is comparable to infant health outcomes in Nepal as a whole. For example, the neonatal mortality rate is our study (32 deaths per 1000 live births) was quite similar to the neonatal mortality for Nepal as a whole during a similar period (33 deaths per 1000 live births) [47]. The living conditions in Sarlahi, which is near sea level and on the border of India, are comparable to those of substantial proportions of populations living in the Indian subcontinent [12].

## Conclusion

Ecological association studies such as this provide a guide to more definitive studies in the search for causal factors in adverse birth outcomes. One way to evaluate these potential seasonal risk factors in Nepal would be to intervene and to assess whether a specific intervention reduces the seasonality of the adverse birth outcomes. For example, the government of Nepal is now encouraging antenatal visits and facility deliveries with a conditional cash transfer program [48]. The expected uptake in antenatal visits is a good opportunity to intervene on nutritional status during pregnancy and to treat women for various morbidities that might reduce the prevalence of adverse birth outcomes. Maternal influenza infections could be reduced with a vaccination program. If food insecurity is related to poor outcomes then maternal nutrition supplementation could be targeted during the months with highest food insecurity.

## Electronic supplementary material

Additional file 1: **STROBE Statement checklist.** (DOC 85 KB)

Additional file 2: Table S1: Stillbirths, Early Neonatal, Perinatal, and Neonatal Mortality Rates and Numbers by Month. (DOCX 304 KB)

Additional file 3: Table S2: Low Birth weight Category by Month. (DOCX 95 KB)

Additional file 4: Figure S1: Mean Birthweight by Sex and Month. (DOCX 53 KB)

Additional file 5: Table S3: Preterm Category by Month. (DOCX 90 KB)

Additional file 6: Figure S2: Average Gestational Age by Month. (DOCX 50 KB)

Additional file 7: Table S4: Small for Gestational Age by Month. (DOCX 93 KB)

Additional file 8: Table S5: Small for Gestational Age & Preterm by Month. (DOCX 89 KB)

Additional file 9: Figure S3: Percent Small for Gestational Age and Preterm by Month. (DOCX 61 KB)

Additional file 10: Figure S4: Food Insecurity by Month (From NNIPS cohort September 2006 – March 2008). (DOCX 49 KB)

Additional file 11: Figure S5: Average Max and Min Temperatures. (DOCX 54 KB)

Additional file 12: Table S6: Summary of Monthly Prevalence of Neonatal Outcomes Peaks and Troughs and Risk Factor Peaks. (DOCX 185 KB)

Additional file 13: Table S7: Logistic Regression for Main Outcomes. (DOCX 467 KB)

Additional file 14: Table S8: Comparison of Characteristics between those with birthweights and those with birthweights missing/collected >72 hours. (DOCX 62 KB)
