# Supplementary material for: Seasonality of birth outcomes in rural Sarlahi District, Nepal: a population-based prospective cohort
Source: BMC Pregnancy Childbirth. 2014 Sep 6;14:310. doi: 10.1186/1471-2393-14-310 (PMC4162951; doi:10.1186/1471-2393-14-310)
Supplement: Supplementary file 3 — Additional file 3: Table S2: Low Birth weight Category by Month. (DOCX 95 KB) [file 12884_2014_1179_MOESM3_ESM.docx]

| **Table 2 - Percent Low Birthweight Category by Month** | | | | | | | |
| --- | --- | --- | --- | --- | --- | --- | --- |
|  | **All Births** | **Very Low Birthweight <2000 g** | | | **Low Birthweight <2500 g** | | |
|  | **Number** | **Number** | **Percentage** | **95% CI** | **Number** | **Percentage** | **95% CI** |
| **January** | 1812 | 71 | 3.9 | 3.1 - 4.9 | 454 | 25.1 | 23.1 - 27.2 |
| **February** | 1093 | 32 | 2.9 | 2.0 - 4.1 | 245 | 22.4 | 20.0 - 25.0 |
| **March** | 1389 | 61 | 4.4 | 3.4 - 5.6 | 359 | 25.8 | 23.6 - 28.2 |
| **April** | 1210 | 66 | 5.5 | 4.2 - 6.9 | 365 | 30.2 | 27.6 - 32.8 |
| **May** | 1200 | 62 | 5.2 | 4.0 - 6.6 | 345 | 28.8 | 26.2 - 31.4 |
| **June** | 1263 | 69 | 5.5 | 4.3 - 6.9 | 365 | 28.9 | 26.4 - 31.5 |
| **July** | 1580 | 93 | 5.9 | 4.8 - 7.2 | 485 | 30.7 | 28.4 - 33.0 |
| **August** | 1927 | 98 | 5.1 | 4.2 - 6.2 | 665 | 34.5 | 32.4 - 36.7 |
| **September** | 2490 | 159 | 6.4 | 5.5 - 7.4 | 844 | 33.9 | 32.0 - 35.8 |
| **October** | 2143 | 124 | 5.8 | 4.8 - 6.9 | 735 | 34.3 | 32.3 - 36.4 |
| **November** | 2086 | 110 | 5.3 | 4.4 - 6.3 | 708 | 33.9 | 31.9 - 36.0 |
| **December** | 2029 | 89 | 4.4 | 3.5 - 5.4 | 593 | 29.2 | 27.3 - 31.3 |
| **Total** | **20222** | **1034** | **5.1** | **4.8 - 5.4** | **6163** | **30.5** | **29.9 - 31.1** |
