# Supplementary material for: Seasonality of birth outcomes in rural Sarlahi District, Nepal: a population-based prospective cohort
Source: BMC Pregnancy Childbirth. 2014 Sep 6;14:310. doi: 10.1186/1471-2393-14-310 (PMC4162951; doi:10.1186/1471-2393-14-310)
Supplement: Supplementary file 4 — Additional file 4: Figure S1: Mean Birthweight by Sex and Month. (DOCX 53 KB) [file 12884_2014_1179_MOESM4_ESM.docx]

Figure 1: Mean Birthweight by Sex and Month
