# Supplementary material for: Seasonality of birth outcomes in rural Sarlahi District, Nepal: a population-based prospective cohort
Source: BMC Pregnancy Childbirth. 2014 Sep 6;14:310. doi: 10.1186/1471-2393-14-310 (PMC4162951; doi:10.1186/1471-2393-14-310)
Supplement: Supplementary file 5 — Additional file 5: Table S3: Preterm Category by Month. (DOCX 90 KB) [file 12884_2014_1179_MOESM5_ESM.docx]

| **Table 3 - Preterm Category by Month** | | | | | | | |
| --- | --- | --- | --- | --- | --- | --- | --- |
|  | **All Births** | **Very Preterm (<34 weeks)** | | | **Preterm (<37 weeks)** | | |
|  | **Number** | **Number** | **Percentage** | **95% CI** | **Number** | **Percentage** | **95% CI** |
| **January** | 1946 | 27 | 1.4 | 0.9 - 2.0 | 346 | 17.8 | 16.1 - 19.6 |
| **February** | 1303 | 15 | 1.2 | 0.6 - 1.9 | 212 | 16.3 | 14.3 - 18.4 |
| **March** | 1529 | 20 | 1.3 | 0.8 - 2.0 | 266 | 17.4 | 15.5 - 19.4 |
| **April** | 1506 | 38 | 2.5 | 1.8 - 3.5 | 278 | 18.5 | 16.5 - 20.5 |
| **May** | 1356 | 25 | 1.8 | 1.2 - 2.7 | 195 | 14.4 | 12.6 - 16.4 |
| **June** | 1394 | 24 | 1.7 | 1.1 - 2.6 | 225 | 16.1 | 14.2 - 18.2 |
| **July** | 1774 | 33 | 1.9 | 1.3 - 2.6 | 286 | 16.1 | 14.4 - 17.9 |
| **August** | 2129 | 48 | 2.3 | 1.7 - 3.0 | 348 | 16.3 | 14.8 - 18.0 |
| **September** | 2764 | 68 | 2.5 | 1.9 - 3.1 | 588 | 21.3 | 19.8 - 22.8 |
| **October** | 3013 | 114 | 3.8 | 3.1 - 4.5 | 680 | 22.6 | 21.1 - 24.1 |
| **November** | 2662 | 58 | 2.2 | 1.7 - 2.8 | 521 | 19.6 | 18.1 - 21.1 |
| **December** | 2286 | 28 | 1.2 | 0.8 - 1.8 | 375 | 16.4 | 14.9 - 18.0 |
| **Total** | **23662** | **498** | **2.1** | **1.9 - 2.3** | **4320** | **18.3** | **17.8 - 18.8** |
