# Supplementary material for: Seasonality of birth outcomes in rural Sarlahi District, Nepal: a population-based prospective cohort
Source: BMC Pregnancy Childbirth. 2014 Sep 6;14:310. doi: 10.1186/1471-2393-14-310 (PMC4162951; doi:10.1186/1471-2393-14-310)
Supplement: Supplementary file 6 — Additional file 6: Figure S2: Average Gestational Age by Month. (DOCX 50 KB) [file 12884_2014_1179_MOESM6_ESM.docx]

Figure 2: Average Gestational Age by Month
