# Supplementary material for: Seasonality of birth outcomes in rural Sarlahi District, Nepal: a population-based prospective cohort
Source: BMC Pregnancy Childbirth. 2014 Sep 6;14:310. doi: 10.1186/1471-2393-14-310 (PMC4162951; doi:10.1186/1471-2393-14-310)
Supplement: Supplementary file 7 — Additional file 7: Table S4: Small for Gestational Age by Month. (DOCX 93 KB) [file 12884_2014_1179_MOESM7_ESM.docx]

| **Table 4 - Small for Gestational Age by Month** | | | | | | | |
| --- | --- | --- | --- | --- | --- | --- | --- |
|  | **All Births** | **SGA <10%** | | | **SGA <3%** | | |
|  | **Number** | **Number** | **Percentage** | **95% CI** | **Number** | **Percentage** | **95% CI** |
| **January** | 1812 | 888 | 49.0 | 46.7 - 51.3 | 448 | 24.7 | 22.8 - 26.8 |
| **February** | 1093 | 490 | 44.8 | 41.9 - 47.8 | 243 | 22.2 | 19.8 - 24.8 |
| **March** | 1389 | 672 | 48.4 | 45.7 - 51.1 | 377 | 27.1 | 24.8 - 29.6 |
| **April** | 1210 | 616 | 50.9 | 48.1 - 53.8 | 364 | 30.1 | 27.5 - 32.8 |
| **May** | 1200 | 645 | 53.8 | 50.9 - 56.6 | 379 | 31.6 | 29.0 - 34.3 |
| **June** | 1262 | 703 | 55.7 | 52.9 - 58.5 | 405 | 32.1 | 29.5 - 34.7 |
| **July** | 1577 | 891 | 56.5 | 54.0 - 59.0 | 519 | 32.9 | 30.6 - 35.3 |
| **August** | 1927 | 1081 | 56.1 | 53.9 - 58.3 | 640 | 33.2 | 31.1 - 35.4 |
| **September** | 2488 | 1320 | 53.1 | 51.1 - 55.0 | 731 | 29.4 | 27.6 - 31.2 |
| **October** | 2141 | 1121 | 52.4 | 50.2 - 54.5 | 661 | 30.9 | 28.9 - 32.9 |
| **November** | 2085 | 1175 | 56.4 | 54.2 - 58.5 | 681 | 32.7 | 30.7 - 34.7 |
| **December** | 2028 | 1048 | 51.7 | 49.5 - 53.9 | 589 | 29.0 | 27.1 - 31.1 |
| **Total** | **20212** | **10650** | **52.7** | **52.0 - 53.4** | **6037** | **29.9** | **29.2 - 30.5** |
