# Supplementary material for: Seasonality of birth outcomes in rural Sarlahi District, Nepal: a population-based prospective cohort
Source: BMC Pregnancy Childbirth. 2014 Sep 6;14:310. doi: 10.1186/1471-2393-14-310 (PMC4162951; doi:10.1186/1471-2393-14-310)
Supplement: Supplementary file 8 — Additional file 8: Table S5: Small for Gestational Age & Preterm by Month. (DOCX 89 KB) [file 12884_2014_1179_MOESM8_ESM.docx]

| **Table 5 - Small for Gestational Age and Preterm by Month** | | | | | | | |
| --- | --- | --- | --- | --- | --- | --- | --- |
|  | **All Births** | **SGA <10% and Preterm** | | | **SGA <3% and Preterm** | | |
|  | **Number** | **Number** | **Percentage** | **95% CI** | **Number** | **Percentage** | **95% CI** |
| **January** | 1812 | 67 | 3.7 | 2.9 - 4.7 | 22 | 1.2 | 0.8 - 1.8 |
| **February** | 1093 | 43 | 3.9 | 2.9 - 5.3 | 4 | 0.4 | 0.1 - 0.9 |
| **March** | 1389 | 52 | 3.7 | 2.9 - 4.9 | 19 | 1.4 | 0.8 - 2.1 |
| **April** | 1210 | 51 | 4.2 | 3.2 - 5.5 | 18 | 1.5 | 0.9 - 2.3 |
| **May** | 1200 | 38 | 3.2 | 2.3 - 4.3 | 11 | 0.9 | 0.5 - 1.6 |
| **June** | 1262 | 50 | 4.0 | 3.0 - 5.2 | 17 | 1.3 | 0.8 - 2.2 |
| **July** | 1577 | 74 | 4.7 | 3.7 - 5.9 | 31 | 2.0 | 1.3 - 2.8 |
| **August** | 1927 | 79 | 4.1 | 3.3 - 5.1 | 24 | 1.2 | 0.8 - 1.8 |
| **September** | 2488 | 137 | 5.5 | 4.6 - 6.5 | 42 | 1.7 | 1.2 - 2.3 |
| **October** | 2141 | 128 | 6.0 | 5.0 - 7.1 | 40 | 1.9 | 1.3 - 2.5 |
| **November** | 2085 | 99 | 4.7 | 3.9 - 5.8 | 33 | 1.6 | 1.1 - 2.2 |
| **December** | 2028 | 82 | 4.0 | 3.2 - 5.0 | 25 | 1.2 | 0.8 - 1.8 |
| **Total** | **20212** | **900** | **4.5** | **4.2 - 4.8** | **286** | **1.4** | **1.3 - 1.6** |
