# Supplementary material for: Seasonality of birth outcomes in rural Sarlahi District, Nepal: a population-based prospective cohort
Source: BMC Pregnancy Childbirth. 2014 Sep 6;14:310. doi: 10.1186/1471-2393-14-310 (PMC4162951; doi:10.1186/1471-2393-14-310)
Supplement: Supplementary file 9 — Additional file 9: Figure S3: Percent Small for Gestational Age and Preterm by Month. (DOCX 61 KB) [file 12884_2014_1179_MOESM9_ESM.docx]

Figure 3: Percent Small for Gestational Age and Preterm by Month
