# Supplementary material for: Seasonality of birth outcomes in rural Sarlahi District, Nepal: a population-based prospective cohort
Source: BMC Pregnancy Childbirth. 2014 Sep 6;14:310. doi: 10.1186/1471-2393-14-310 (PMC4162951; doi:10.1186/1471-2393-14-310)
Supplement: Supplementary file 10 — Additional file 10: Figure S4: Food Insecurity by Month (From NNIPS cohort September 2006 – March 2008). (DOCX 49 KB) [file 12884_2014_1179_MOESM10_ESM.docx]

Figure 4: Food Insecurity by Month (From NNIPS cohort September 2006 – March 2008)
