# Supplementary material for: Seasonality of birth outcomes in rural Sarlahi District, Nepal: a population-based prospective cohort
Source: BMC Pregnancy Childbirth. 2014 Sep 6;14:310. doi: 10.1186/1471-2393-14-310 (PMC4162951; doi:10.1186/1471-2393-14-310)
Supplement: Supplementary file 14 — Additional file 14: Table S8: Comparison of Characteristics between those with birthweights and those with birthweights missing/collected >72 hours. (DOCX 62 KB) [file 12884_2014_1179_MOESM14_ESM.docx]

| **Table 8 - Comparison of Characteristics between those with birthweights and those with birthweights missing/collected >72 hours** | | | |
| --- | --- | --- | --- |
| **Variable** | **Weight <72 hours** | **Weight missing** | **p-value*** |
| Literate | 25% | 30% | 0.001 |
| Female | 49% | 47% | 0.082 |
| Nulliparous | 25% | 31% | <0.001 |
| Pahadi | 29% | 28% | 0.637 |
| Highest SES Q. | 24% | 28% | <0.001 |
| 2nd Q. | 21% | 21% |  |
| 3rd Q. | 22% | 20% |  |
| Lowest Q. | 33% | 31% |  |
| Mean Gestational Age | 39.3 (sd=2.4) | 38.9 (sd=3.2) | <0.001 |
| Mean Maternal Age | 23.9 (sd=5.3) | 23.5 (sd=5.1) | <0.001 |
| *P-value based on Pearson's chi-square (categorical) and t-test (continuous) | | | |
