# Supplementary material for: Seasonality of birth outcomes in rural Sarlahi District, Nepal: a population-based prospective cohort
Source: BMC Pregnancy Childbirth. 2014 Sep 6;14:310. doi: 10.1186/1471-2393-14-310 (PMC4162951; doi:10.1186/1471-2393-14-310)
Supplement: Supplementary file 12 [file 12884_2014_1179_MOESM12_ESM.docx]

Table 6 - Summary of Monthly Prevalence of Neonatal Outcomes Peaks and Troughs and Risk Factor Peaks

	January	February	March	April	May	June	July	August	September	October	November	December
**Stillbirth Rate**												
**Perinatal Mortality Rate**												
**Neonatal Mortality Rate**												
**Low Birthweight**												
**Preterm**												
**Small-for-Gestational Age**												
**Preterm and Small-for-Gestational Age**												
**Temperature**												
**Food Insecurity**												
**Anemia**												
**Influenza**												
**RSV**												

*Red shading indicates a peak period prevalence and green shading represents a low period prevalence
