# Supplementary material for: Seasonality of birth outcomes in rural Sarlahi District, Nepal: a population-based prospective cohort
Source: BMC Pregnancy Childbirth. 2014 Sep 6;14:310. doi: 10.1186/1471-2393-14-310 (PMC4162951; doi:10.1186/1471-2393-14-310)
Supplement: Supplementary file 13 [file 12884_2014_1179_MOESM13_ESM.docx]

Table 7 - Logistic Regression for Main Outcomes

	Unadjusted			Adjusted*		
**Preterm**						
	**Odds Ratio**	**P>lzl**	**95% Conf. Interval**	**Odds Ratio**	**P>lzl**	**95% Conf. Interval**
**January**	1 (ref)			1 (ref)		
**February**	0.90	0.26	0.74 - 1.08	0.89	0.22	0.73 - 1.07
**March**	0.97	0.77	0.82 - 1.16	0.94	0.50	0.78 - 1.13
**April**	1.05	0.61	0.88 - 1.25	0.99	0.95	0.83 - 1.19
**May**	0.78	0.01	0.64 - 0.94	0.74	0.00	0.61 - 0.90
**June**	0.89	0.22	0.74 - 1.07	0.90	0.26	0.74 - 1.08
**July**	0.89	0.18	0.75 - 1.06	0.90	0.23	0.75 - 1.07
**August**	0.90	0.22	0.77 - 1.06	0.86	0.08	0.73 - 1.02
**September**	1.25	0.00	1.08 - 1.45	1.17	0.05	1.00 - 1.36
**October**	1.35	0.00	1.17 - 1.56	1.26	0.00	1.09 - 1.46
**November**	1.13	0.12	0.97 - 1.31	1.06	0.44	0.91 - 1.24
**December**	0.91	0.24	0.77 - 1.07	0.88	0.11	0.74 - 1.03
**Low Birthweight**						
	**Unadjusted**			**Adjusted**		
	**Odds Ratio**	**P>lzl**	**95% Conf. Interval**	**Odds Ratio**	**P>lzl**	**95% Conf. Interval**
**January**	1 (ref)			1 (ref)		
**February**	0.86	0.11	0.72 - 1.03	0.85	0.08	0.71 - 1.02
**March**	1.04	0.61	0.89 - 1.22	1.04	0.61	0.88 - 1.23
**April**	1.29	0.00	1.10 - 1.52	1.28	0.01	1.08 - 1.51
**May**	1.21	0.03	1.02 - 1.42	1.22	0.02	1.03 - 1.44
**June**	1.22	0.02	1.03 - 1.43	1.27	0.01	1.07 - 1.50
**July**	1.32	0.00	1.14 - 1.54	1.39	0.00	1.19 - 1.63
**August**	1.58	0.00	1.37 - 1.82	1.58	0.00	1.36 - 1.83
**September**	1.53	0.00	1.34 - 1.76	1.51	0.00	1.31 - 1.74
**October**	1.56	0.00	1.36 - 1.79	1.51	0.00	1.31 - 1.75
**November**	1.54	0.00	1.34 - 1.77	1.48	0.00	1.28 - 1.71
**December**	1.24	0.00	1.07 - 1.43	1.19	0.02	1.03 - 1.38
**Neonatal Mortality**						
	**Unadjusted**			**Adjusted**		
	**Odds Ratio**	**P>lzl**	**95% Conf. Interval**	**Odds Ratio**	**P>lzl**	**95% Conf. Interval**
**January**	1 (ref)			1 (ref)		
**February**	0.94	0.78	0.62 - 1.44	0.85	0.46	0.54 - 1.31
**March**	0.73	0.16	0.47 - 1.13	0.74	0.17	0.48 - 1.14
**April**	1.21	0.33	0.83 - 1.77	1.17	0.44	0.79 - 1.72
**May**	1.35	0.13	0.92 - 1.97	1.32	0.16	0.90 - 1.95
**June**	1.21	0.34	0.82 - 1.78	1.25	0.27	0.84 - 1.85
**July**	1.16	0.43	0.80 - 1.68	1.16	0.44	0.80 - 1.68
**August**	1.39	0.06	0.99 - 1.96	1.37	0.08	0.97 - 1.93
**September**	1.14	0.44	0.82 - 1.60	1.09	0.64	0.77 - 1.53
**October**	1.28	0.14	0.93 - 1.77	1.22	0.23	0.88 - 1.70
**November**	0.86	0.39	0.60 - 1.22	0.80	0.24	0.56 - 1.16
**December**	0.91	0.61	0.63 - 1.31	0.88	0.50	0.61 - 1.28
**Small for Gestational Age (<10%)**						
	**Unadjusted**			**Adjusted**		
	**Odds Ratio**	**P>lzl**	**95% Conf. Interval**	**Odds Ratio**	**P>lzl**	**95% Conf. Interval**
**January**	1 (ref)			1 (ref)		
**February**	0.85	0.03	0.73 - 0.98	0.82	0.01	0.70 - 0.95
**March**	0.98	0.73	0.85 - 1.12	0.96	0.55	0.83 - 1.10
**April**	1.08	0.31	0.93 - 1.25	1.08	0.33	0.93 - 1.25
**May**	1.21	0.01	1.04 - 1.40	1.22	0.01	1.05 - 1.42
**June**	1.31	0.00	1.13 - 1.51	1.33	0.00	1.15 - 1.54
**July**	1.35	0.00	1.18 - 1.55	1.36	0.00	1.18 - 1.56
**August**	1.33	0.00	1.17 - 1.51	1.32	0.00	1.16 - 1.51
**September**	1.18	0.01	1.04 - 1.33	1.17	0.01	1.04 - 1.33
**October**	1.14	0.04	1.01 - 1.30	1.12	0.08	0.99 - 1.28
**November**	1.34	0.00	1.18 - 1.52	1.31	0.00	1.16 - 1.50
**December**	1.11	0.10	0.98 - 1.26	1.08	0.26	0.95 - 1.23

***Adjusted for infant sex, maternal literacy, parity, ethnicity, and socioeconomic status**
