# Supplementary material for: Seasonality of birth outcomes in rural Sarlahi District, Nepal: a population-based prospective cohort
Source: BMC Pregnancy Childbirth. 2014 Sep 6;14:310. doi: 10.1186/1471-2393-14-310 (PMC4162951; doi:10.1186/1471-2393-14-310)
Supplement: Supplementary file 14 [file 12884_2014_1179_MOESM2_ESM.docx]

Table 1 - Stillbirths, Early Neonatal, Perinatal, and Neonatal Mortality Rates and Numbers by Month													
		Stillbirth			Early Neonatal Mortality			Perinatal Mortality			Neonatal Mortality		
	Live births	Rate	95% CI	Number	Rate	95% CI	Number	Rate	95% CI	Number	Rate	95% CI	Number
**January**	1946	33.3	25.9 - 42.1	67	23.1	17.0 - 31.0	45	55.6	46.0 - 66.6	112	29.3	22.2 - 37.8	57
**February**	1303	51.0	40.0 - 64.0	70	23.8	16.3 - 33.7	31	73.6	60.3 - 88.7	101	27.6	19.4 - 38.1	36
**March**	1529	42.0	32.7 - 53.0	67	14.4	9.1 - 21.9	22	55.8	45.0 - 68.2	89	21.6	14.9 - 30.2	33
**April**	1506	44.4	34.8 - 55.8	70	29.9	22.0 - 40.0	45	73.0	60.6 - 86.9	115	35.2	26.5 - 45.8	53
**May**	1356	45.1	34.9 - 57.2	64	31.7	23.2 - 42.8	43	75.4	62.2 - 90.3	107	39.1	29.4 - 50.8	53
**June**	1394	29.9	21.7 - 40.1	43	28.0	20.1 - 38.3	39	57.1	45.6 - 70.3	82	35.2	26.1 - 46.2	49
**July**	1774	30.1	22.7 - 39.0	55	29.9	22.6 - 39.1	53	59.0	48.7 - 70.9	108	33.8	25.9 - 43.3	60
**August**	2129	44.4	36.3 - 53.9	99	33.3	26.3 - 42.2	71	76.3	65.6 - 88.1	170	40.4	32.4 - 49.7	86
**September**	2764	36.9	30.4 - 44.5	106	27.1	21.5 - 34.1	75	63.1	54.5 - 72.6	181	33.3	26.9 - 40.7	92
**October**	3013	27.8	22.2 - 34.2	86	28.5	23.1 - 35.5	86	55.5	47.7 - 64.2	172	37.2	30.7 - 44.6	112
**November**	2662	35.2	28.6 - 42.7	97	14.7	10.5 - 20.2	39	49.3	34.2 - 49.4	136	25.2	19.6 - 31.9	67
**December**	2286	29.3	22.9 - 36.9	69	19.7	14.5 - 26.4	45	48.4	22.9 - 36.9	114	26.7	20.5 - 34.1	61
**Total**	**23662**	**36.4**	**34.1 - 38.8**	**893**	**25.1**	**23.3 - 27.4**	**594**	**60.6**	**57.6 - 63.6**	**1487**	**32.1**	**29.9 - 34.4**	**759**
